# Detection of plasma *EGFR* mutations for personalized treatment of lung cancer patients without pathologic diagnosis

**DOI:** 10.1002/cam4.2869

**Published:** 2020-01-28

**Authors:** Qinfang Deng, Qiyu Fang, Hui Sun, Aditi P. Singh, Mariam Alexander, Shenduo Li, Haiying Cheng, Songwen Zhou

**Affiliations:** ^1^ Department of Oncology The Third Affiliated Hospital of Soochow University Changzhou China; ^2^ Department of Oncology Shanghai Pulmonary Hospital Tongji University School of Medicine Shanghai China; ^3^ Department of Oncology Montefiore Medical Center Albert Einstein College of Medicine Bronx NY USA; ^4^ Department of Medicine Jacobi Medical Center Bronx NY USA

**Keywords:** ctDNA, *EGFR* mutations, lung cancer, personalized treatment

## Abstract

**Introduction:**

Next‐generation sequencing (NGS) and digital polymerase chain reaction (PCR) based platforms have been used to detect *EGFR* mutations in plasma circulating tumor DNA (ctDNA) with high accuracy. Generally, molecular testing is performed after histopathological analysis. However, many patients with suspected advanced nonsmall cell lung cancer are unable to undergo biopsy thus forgoing potential treatment with highly effective tyrosine kinase inhibitors (TKIs) in patients with sensitizing *EGFR* mutations. We examined the utility of ctDNA testing to detect *EGFR* mutations in patients' plasma, where tissue biopsy is not feasible.

**Methods:**

We conducted a single‐center, prospective study of 30 Chinese patients with suspected advanced lung cancer, who were unable to undergo a biopsy for initial diagnosis due to comorbidities or poor performance status. Patients with plasma *EGFR* sensitizing mutations were treated with first‐generation EGFR TKIs.

**Results:**

Twenty of 30 patients enrolled had sensitizing *EGFR* mutations in ctDNA and were started on EGFR TKIs. After a median follow‐up of 12 months, median progression‐free survival (PFS) was 10 months and median overall survival (OS) was not reached. The median OS for the 10 untreated patients was 3 months.

**Conclusions:**

In our study, patients with plasma *EGFR* mutations treated with TKIs showed disease control rate (DCR) and PFS similar to historical controls that were treated based on tissue testing. This is the first prospective study showing that ctDNA genotyping provides a feasible diagnostic approach for frail lung cancer patients who are unable to undergo biopsy, which subsequently leads to EGFR‐targeted therapy, and improved outcomes in this subgroup of patients.

## Graphical abstract

This study is the first to prospectively evaluate the utility and feasibility of plasma circulating tumor DNA to detect *EGFR* mutations in frail patients with lung cancer, when tissue biopsy was not feasible. Incorporating plasma molecular profiling with clinical data allowed patients with sensitizing *EGFR* mutations to undergo treatment with highly effective tyrosine kinase inhibitors in a timely manner and gain significant improvement in overall survival, disease control rate, and progression free survival.

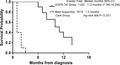

## 1 INTRODUCTION

Lung cancer is the most common cancer as well as the leading cause of cancer related deaths worldwide, with 2.09 million new cases and 1.76 million deaths in 2018.^1^ Nonsmall cell lung cancer (NSCLC) accounts for 85% of all lung cancer cases and almost two‐thirds have distant metastases at the time of diagnosis.^2^ Molecularly targeted agents against oncogenic drivers like *EGFR*, *ALK*, and *ROS1* have revolutionized the treatment paradigm for a subset of NSCLC patients with these genomic alterations. The National Comprehensive Cancer Network (NCCN) guidelines recommend molecular genotyping of all nonsquamous cell lung carcinomas as well as for squamous cell lung cancers in nonsmokers, with small biopsies or mixed histology.^3^

Traditionally, molecular genotyping has been performed on tumor tissue. With advances in testing platforms, we can now detect these alterations in circulating tumor DNA (ctDNA) in patients' plasma with high accuracy, using next‐generation sequencing (NGS) or digital polymerase chain reaction (PCR) assays.^4^ Despite these advances, tissue biopsy and tumor genotyping remains the gold standard for initial diagnosis of patients with molecular genotyping conducted after histopathological diagnosis. This is done not only to delineate key pathologic features like histology and immune microenvironment, but also due to the approximately 30% false negative rate of detecting these mutations in ctDNA.^5^

However, a significant proportion of patients are unable to tolerate invasive procedures like tissue biopsies to establish pathologic diagnosis due to poor performance status or comorbidities resulting in elevated risk from anesthesia and surgery. Treatment for these patients is often delayed or limited due to lack of definitive diagnoses. The NCCN guidelines recommend plasma genotyping in the event that sufficient tissue is not available, both at initial diagnosis as well as at disease progression.^3^ However, the application of ctDNA for detecting *EGFR* mutations at initial diagnosis in patients without pathologic confirmation of lung cancer has not been well established. In this study, we prospectively explored the role of ctDNA in detecting *EGFR* mutations in patients' plasma and determined the efficacy of EGFR tyrosine kinase inhibitors (TKIs) in patients with suspected advanced lung cancer, in the absence of pathologic diagnosis. We hypothesized that this noninvasive approach would be beneficial for patients with poor performance statuses (ECOG 3 ~ 4), patients who were unable to undergo invasive procedures like a bronchoscopy or image‐guided needle biopsies due to medical comorbidities or due to inaccessible lesions, and patients who have had nondiagnostic biopsies.

## 2 METHODS

### 2.1 Study subjects

We conducted a single‐center, prospective study in the Department of Oncology at Shanghai Pulmonary Hospital from August 1, 2016 to September 1, 2017. Thirty patients with suspected advanced lung cancer who were unable to tolerate tissue biopsy (due to comorbidities or poor performance status) were screened for the study and subsequently enrolled. Inclusion criteria included patients with clinical symptoms suggestive of primary lung cancer or its distant metastasis; whole body PET‐CT with FDG‐avid lung mass with metastatic lesions in the mediastinum, contralateral lung, or distant organs; an Eastern Cooperative Oncology Group (ECOG) performance status (PS) of 3 or 4, a negative serum T‐SPOT^®^ (Oxford Immunotec Inc) test to rule out tuberculosis; and at least one elevated tumor marker out of a panel recommended by the Chinese guidelines for lung cancer diagnosis.^6^, ^7^ Although patients were excluded from the study if they had primary co‐occurring malignancy, an occult malignancy could not be completely ruled out. Patients were required to undergo PET‐CT scans, serum tumor marker analysis, and brain MRI. Plasma genotyping by NGS was funded by a precision treatment research grant. The clinical investigation ethics committee of the Shanghai Pulmonary Hospital approved the study and all participants signed voluntary informed consents.

### 2.2 Blood sample collection and extraction of ctDNA

Next‐generation sequencing (NGS) or Amplification Refractory Mutation System (ARMS) were used to identify *EGFR* mutations in ctDNA and only plasma was used for cell‐free DNA (cfDNA) extraction. NGS was the preferred method, but some samples underwent ARMS due to the prohibitive cost of NGS.

Ten milliliters (mL) of fasting peripheral venous blood was collected in blood collection tubes (BCT) (Streck). Samples were transported at room temperature and processed within 48 hours of blood draw, according to the Streck BCT protocol at Shanghai SmartQuerier Biomedicine Co. Ltd. Samples were centrifuged at 2500 g for 10 minutes. The serum supernatant was then collected and centrifuged at 16 000 g for 10 minutes. The resultant 3‐5 mL of supernatant was collected and circulating free DNA was extracted using QIAamp Circulating Nucleic Acid Kit (Qiagen) for NGS or using DNeasy Blood & Tissue Kit (Qiagen) for ARMS. Germline DNA was extracted from the supernatant after the first centrifuge using QIAamp DNA Blood Mini Kit (Qiagen). All patient blood samples required two NGS sequencing libraries involving plasma DNA and germline genomic DNA. A minimum of 1 µg of germline DNA was extracted from each sample.

### 2.3 ctDNA sequencing and *EGFR* mutation analysis

Next‐generation sequencing (NGS) or Amplification Refractory Mutation System (ARMS) were used to identify *EGFR* mutations in ctDNA. NGS was the preferred method, but some samples underwent ARMS due to the prohibitive cost of NGS.

### 2.4 Next‐generation sequencing (NGS)

DNA extracted using QIAamp Circulating Nucleic Acid Kit and germline DNA library were used for NGS. The library was constructed using the KAPA kit (Kapa Biosystems). Cell‐free DNA was quantified with Qubit v3.0 (Thermo Fisher Scientific). The first step in library construction was end repair and A‐tailing using magnetic beads, followed by purification using Agencourt AMPure XP beads (Beckman‐Coulter). The adapter ligation reaction was carried out at 16°C for 16 hours (overnight), followed by size selection. The constructed library was then obtained from the linking library after 4 ~ 9 PCR cycles (number of PCR cycles was selected based on the initial amount of input DNA). The size distribution of the constructed library was detected using a 2100 Bioanalyzer (Agilent). The library was enriched by tailor‐made SeqCap EZ Choice Library probes (Roche, NimbleGen). According to the manufacturer's manual, 8 ~ 10 ligated libraries were mixed together and hybridized to enrich target areas. An enriched library was obtained by the captured hybrid library after 12 ~ 14 PCR cycles of amplification. Finally, the library was quantified by qubit v3.0 & KAPA qPCR (Kapa Biosystems), and sequenced with the Illumina HiSeq X10 sequencer. Paired‐end sequencing data were then matched to human hg19 version of the reference genome by MEM command of BWA (V0.7.15‐r1140). The results were sorted and used to generate index files by SAMtools (V1.3).

Quality control of the sequencing data was completed by a self‐built Python script, which included a series of statistical parameters including aligned ratio, aligned quality, sequence quality, capture efficiency, and repetition rate. Bases with quality scores >30 (Q30) were identified for further analysis. Average sequencing depth was ~8000. The gene list is provided in Table S1. One hundred and fifty‐six genes were sequenced. Base substitutions, insertion and deletions (indels), fusions, and copy number variations (CNV) were reported. These variants were called from plasma data while germline data were used to exclude germline variants. Variants with >20% AF in germline were excluded for further study. Variants with >2 deduped reads for hotspots and >5 deduped reads for nonhotspots were defined as positive signals. Base substitutions and indels were further verified by Vardict (V1.4.5), GATK (v3.5‐0‐g36282e4), and Mutect2 through strict postprocessing that could filter out false positive results. Fusion and breakpoint detection were performed by FACTERA (1.4.4) software, and CNV detection was done by CNVkit (V0.8.2).

### 2.5 Amplification Refractory Mutation System (ARMS)

DNA from plasma was extracted using the DNeasy Blood & Tissue Kit (Qiagen). Five genes were sequenced—*EGFR*, *ALK*, *ROS1*, *BRAF*, and *KRAS*. Twenty‐nine types of *EGFR* mutations in exons 18 to 21 were detected using the Human *EGFR* Gene Mutations Fluorescence PCR Diagnostic Kit (Amoy Diagnostics) in accordance with the manufacturer's instructions. Data analysis was conducted using MxPro v4.10 (Stratagene). According to the manufacturer's instructions, positive results were defined as: (a) cycle threshold (Ct) <26 and (b) Ct >26 and ΔCt (difference between the mutation Ct and control Ct) < the cut‐off ΔCt value (for instance, 11 for 19Del and L858R, 7 for 719X, 6.5 for S768I and L861Q).^8^

### 2.6 Data collection and follow‐up

Demographic and clinical data were collected for all patients. Patients were staged using whole body PET‐CT and brain MRI. Patients with suspected bone metastases underwent a spinal MRI. Serum tumor markers, including CEA, NSE, CA211, SCC, and Pro‐GRP were analyzed for all patients. The serum for tumor markers detection was prepared by conventional centrifugation at 3000 rpm. All patients who were treated with EGFR TKIs were followed up 1 month after initiation of treatment, and then every 2 months thereafter. At each visit, they underwent physical examinations and chest CT scans. *EGFR* wild‐type patients were not given any antitumor treatment and received best supportive care due to lack of pathological diagnosis and poor ECOG score. They were followed up once every month.

### 2.7 Response evaluation

Efficacy of treatment was determined by RECIST 1.1 using chest CT scans or PET‐CT scans at follow‐up visits, with response to treatment being determined as complete response (CR), partial response (PR), stable disease (SD), or progressive disease (PD).^9^ The primary endpoint was progression‐free survival (PFS), and secondary endpoints were objective response rate (ORR), disease control rate (DCR), and overall survival (OS).

### 2.8 Statistics analysis

Patient clinical characteristics were analyzed using descriptive statistics and survival analysis was performed using the Kaplan‐Meier method. Statistical significance was defined as *P* < .05. Data were analyzed with SPSS 21.0.

## 3 RESULTS

The baseline characteristics of 30 patients are described in Table 1. Nineteen patients were male and 11 were female. The median age was 66 years (range 37‐85 years). Five patients were above 80 years of age. Twenty‐one patients were smokers, seven patients were nonsmokers, and the smoking status was unknown for two patients. Performance status (PS) was either ECOG PS 3 or PS 4. PET‐CT showed extensive bone metastases in 16 patients with spinal compression symptoms. Thirteen patients had brain metastases and eight had multiple extrapulmonary metastases. At least one serum tumor marker was elevated in all 30 patients. CEA was most frequently increased (76.7%, n = 23). All patients were unable to obtain invasive biopsies due to comorbidities or complications including hemoptysis, respiratory failure, cerebral infarction, and poor pulmonary reserve (multiple pulmonary bullae).

**Table 1:** Patient demographics and baseline clinical characteristics

Characteristics	Treatment group	Untreated group	*P*
Sex			
Male	11	8	.180
Female	9	2	
Smoking history (%)			
Smoker	12	9	.204
Nonsmoker^a^ (or unknown smoking history)	8	1	
Median age (years)	67	66	1.000
ECOG PS			
PS = 3	15	7	1.000
PS = 4	5	3	
Metastatic site			
Bone (spine)	10	6	.816
Brain	9	4	
Extrapulmonary	6	2	
Complications			
Hemoptysis	1	2	
Respiratory failure	1	1	
Comorbidities			
Cerebral infarction	1	1	
Multiple pulmonary bullae	1	2	
Serum tumor markers			
Serum T‐SPOT^®^ negative	20	10	
CEA	15	8	
CA 211	11	3	
CA50	4	1	
CA19‐9	5	2	
NSE	3	1	
CA15‐3	2	2	
CA242	2	1	
Osteocalcin, N‐MID^®^	2	3	
≥3 types	8	3	
ctDNA results			
*EGFR* Exon19 del NGS	5		
*EGFR* Exon19 del ARMS	4		
*EGFR* Exon21 L858R NGS	3		
*EGFR* Exon21 L858R ARMS	7		
Complex mutations NGS	1		

Abbreviations: CA, cancer antigen; CEA, carcinoembryonic antigen; ECOG, Eastern Cooperative Oncology Group; NSE, neuron‐specific enolase.

a Lifetime smoking history of fewer than 100 cigarettes.

Sensitizing *EGFR* mutations were detected in the plasma of 20 patients (Table 1). All 20 patients were started on first‐generation EGFR TKIs (Table 2). Eleven patient samples that were analyzed by ARMS, were all positive for *EGFR* mutations. Nineteen patient samples were assessed by NGS; 9 were positive and 10 were negative for *EGFR* mutations. We performed ddPCR and NGS in six patients simultaneously, and the concordant rate was 100%. Among the patients with *EGFR* mutations, 60% (12/20) were smokers, 30% (6/20) were nonsmokers, and 10% (2/20) had an unknown smoking history. Among patients with no *EGFR* mutations, 90% (9/10) were smokers and 10% (1/0) were nonsmokers. Ten patients without *EGFR* mutations only received the best supportive care. Of them, 90% (9/10) were smokers and 10% (1/0) were nonsmokers. For ctDNA *EGFR* mutations, 9 patients had exon 19 deletions, 10 had Exon 21 L858R mutations, and 1 had a complex mutation, p.T790M (0.438%)/p.G719A (9.927%)/p.L861Q (9.792%)/p747_752del (0.5%) (percentages represent allele fraction).

**Table 2:** Characteristics and clinical outcome of *EGFR*‐mutated patients

Patient	*EGFR* mutation detected	First‐line EGFR TKI	Clinical outcome^a^	PFS (months)	OS (months)	ECOG (prior to treatment)	ECOG (posttreatment)	Follow‐up (months)	Subsequent treatment
1	*EGFR* 21 L858R	Gefitinib	PR (30.3%)	7.00	7.00	4	1	7.00	Gefitinib
2	*EGFR* 19 del	Gefitinib	PR (31.6%)	10.00	12.00	3	2	12.00	Chemotherapy (pemetrexed) + gefitinib
3	*EGFR* 19 del	Gefitinib	PR (50%)	8.00	12.00	3	1	12.00	Chemotherapy (pemetrexed) + gefitinib
4	*EGFR* 21 L858R	Gefitinib	PR (41.9%)	11.00	12.00	3	0	12.00	Chemotherapy (pemetrexed) + gefitinib
5	*EGFR* 19 del	Icotinib	PR (30.7%)	6.00	8.00	3	1	8.00	Chemotherapy (pemetrexed) + icotinib
6	*EGFR* 21 L858R	Icotinib	PR (34.6%)	8.00	9.00	3	1	9.00	Erlotinib due to brain metastases
7	*EGFR* 21 L858R	Gefitinib	PR (47.3%)	10.00	16.00	3	0	16.00	Chemotherapy (pemetrexed/carboplatin) + gefitinib
8	*EGFR* 21 L858R	Icotinib	PR (43.3%)	10.00	10.00	3	1	10.00	Gefitinib
9	*EGFR* 21 L858R	Gefitinib	PR (30%)	14.00	20.00	4	1	20.00	Bone radiotherapy + gefitinib
10	*EGFR* 19 del	Gefitinib	PR (32%)	12.00	12.00	3	0	12.00	Gefitinib
11	*EGFR* 19 del	Gefitinib	PR (31%)	8.00	8.00	3	0	8.00	Gefitinib
12	*EGFR* 21 L858R	Gefitinib	SD (5%)	7.00	12.00	3	1	12.00	Gefitinib
13	*EGFR* 19del	Erlotinib	PR (69.7%)	6.00	17.00	3	1	17.00	Osimertinib
14	*EGFR* p.T790M *EGFR* p.G719A *EGFR* p.L861Q *EGFR* p747_752del	Erlotinib	PR (33%)	12.00	17.00	4	1	17.00	Chemotherapy + erlotinib
15	*EGFR* 21 L858R	Icotinib	SD (10%)	7.00	11.00	3	1	11.00	Osimertinib
16	*EGFR* 21 L858R	Gefitinib	PR (31.5%)	14.00	14.00	4	1	14.00	Gefitinib
17	*EGFR* 19del	Gefitinib	PR (35%)	7.00	7.00	4	1	7.00	Gefitinib
18	*EGFR* 19del	Gefitinib	PR (31%)	6.00	6.00	3	1	6.00	Gefitinib
19	*EGFR* 21 L858R	Gefitinib	PR (39%)	6.00	6.00	3	1	6.00	Gefitinib
20	*EGFR* 19del	Gefitinib	PR (55.8%)	10.00	10.00	3	1	10.00	Gefitinib

Abbreviations: ECOG, Eastern Cooperative Oncology Group; OS, overall survival; PFS, progression‐free survival.

a Maximum reduction from baseline (%).

**Figure 1:**
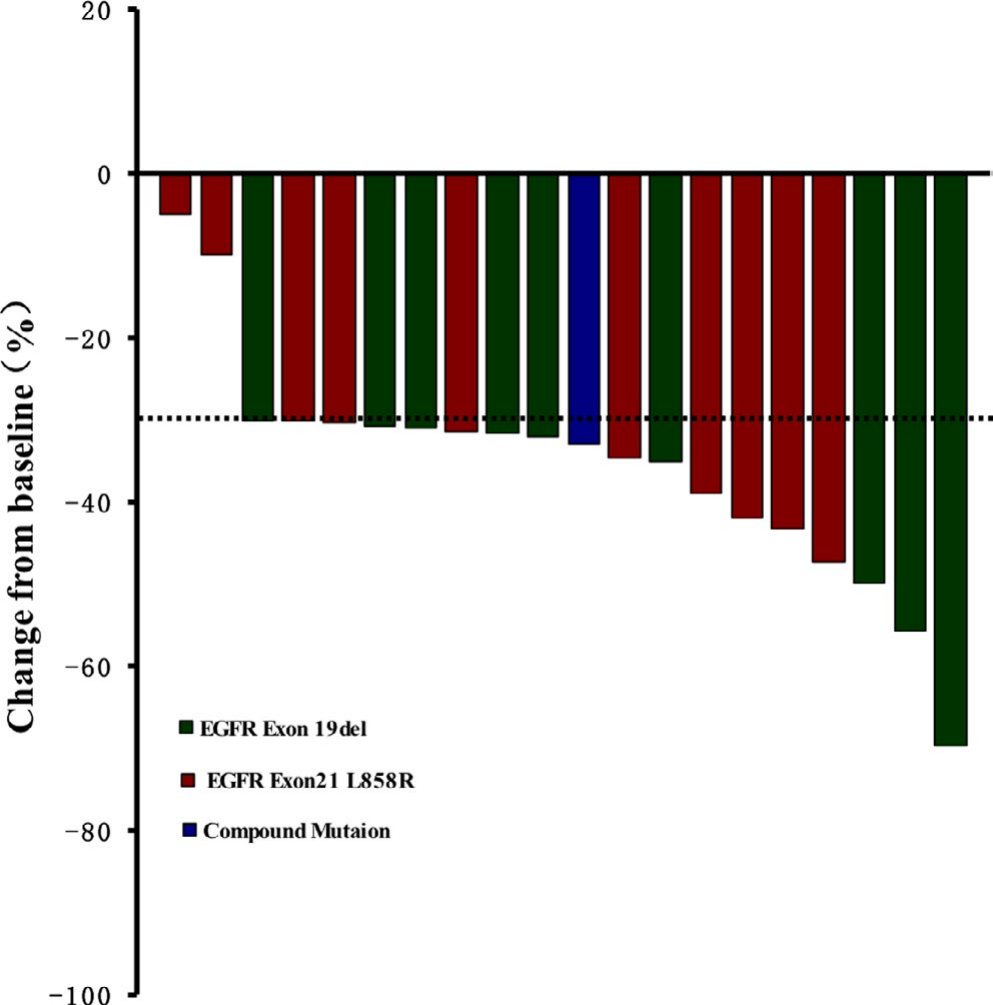
Waterfall of effect after the treatment of *EGFR* tyrosine kinase inhibitor (TKI)

**Figure 2:**
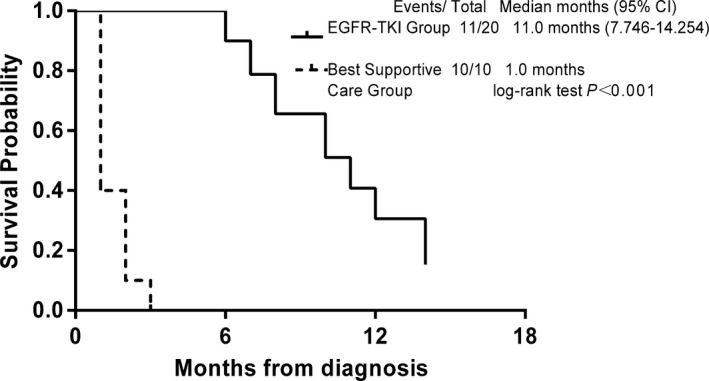
Kaplan‐Meier estimate of progression‐free survival in patients who received *EGFR* tyrosine kinase inhibitors (TKIs)

**Figure 3:**
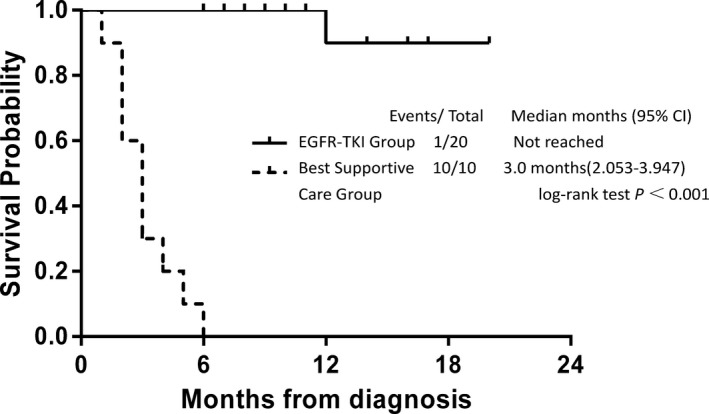
Kaplan‐Meier estimate of overall survival stratified by treatment. TKI, tyrosine kinase inhibito)

One month after EGFR TKIs treatment, PR was observed in 18 patients and SD was observed in 2 patients, resulting in an ORR of 90% (18/20) and a DCR of 100% (20/20) (Figure 1; Table 2). As of February 11, 2018, 11 patients from the treatment group showed PD, including one who died because of discontinuing treatment due to financial hardship (PFS was 10 months; Figure 2). Seven months after first‐generation EGFR‐TKI treatment, one patient presented with disease progression and was found to have the *EGFR* T790M resistance mutation NGS analysis of peripheral blood. The patient was subsequently treated with the third‐generation EGFR TKI (Osimertinib) and achieved a PR 1 month later. The patient had enjoyed SD for 4 months at last follow‐up. After a median follow‐up of 12 months, 18 of 20 patients in the treatment group survived more than 6 months (90%), including 5 cases who survived more than 1 year (25%), and 2 patients who are still on surveillance. Only 1 of 10 patients in the *EGFR* wild‐type untreated group survived 6 months. Median OS of the untreated group was 3 months (95% CI 2.05‐3.9 months), which was significantly worse than the treatment group (*P* < .001; Figure 3). COX regression analysis also demonstrated that only ECOG‐PS after 1 month treatment was significantly associated with OS (univariate analysis: *P* = .036; multivariate analysis: *P* = .044).

With EGFR TKI treatment, all 20 patients were reported to have improved performance status 1 month after starting treatment with TKIs (Table 2). Furthermore, pathology was obtained after improvement of symptoms in nine patients who were able to tolerate lung biopsy or thoracentesis. The pathology reports confirmed adenocarcinoma, four of them underwent detection of mutations in tissue, and consistent with plasma (Table 3). Four of these nine patients received further chemotherapy, one received bone cement treatment, and one received radiation therapy to the bone. On the contrary, patients in the untreated group deteriorated rapidly.

**Table 3:** Reasons why biopsies were not performed in the EGFR TKI‐treated group and best supportive care group

Patient	Age/sex	Smoking status	Reasons	ctDNA detection method	ctDNA results (VAF%)	Gene detection after pathological acquisition^a^
1	63/F	No	Paraplegia due to bone metastases. Lesion not amenable to transthoracic biopsy and lung puncture given anatomic location. Patient unable to tolerate bronchoscopy.	NGS	EGFR21 L858R (15.499%) (ddPCR *EGFR* L858R)	Adenocarcinoma NO detection of gene due to financial condition
2	71/F	Yes	The lesion not amenable to safe biopsy because of proximity to descending aorta. ECOG = 3, unacceptable thoracotomy	NGS	*EGFR* 19del (8.75%)	Adenocarcinoma NGS:*EGFR* 19del
3	54/F	No	Two prior nondiagnostic bronchoscopes and lesion not amenable to transthoracic needle biopsy. Patient unable to tolerate biopsy of lumbar metastasis. Lesions not amenable to lung puncture given anatomic location.	NGS	*EGFR* 19 del (33.25%) (ddPCR *EGFR* 19 746‐750 del)	Adenocarcinoma NO detection of gene due to financial condition
4	51/M	Yes	Poor performance status precluded safe bronchoscopy and thoracotomy.	NGS	*EGFR* 21 L858R (0.475%) (ddPCR *EGFR* L858R)	Adenocarcinoma NO detection of gene due to financial condition
5	70/M	Yes	Respiratory failure, multiple brain metastases, accompanied by intracranial hypertension, and intolerance to bronchoscopy. Lesions not amenable to lung puncture given anatomic location.	NGS	*EGFR* 19 del (0.323333%) (ddPCR *EGFR* 19 746‐750 del)	Adenocarcinoma NO detection of gene due to financial condition
6	83/M	Yes	Lesions not amenable to lung puncture because of anatomic location. The patient was 83 y old with multiple brain metastases, accompanied by intracranial hypertension and intolerance to bronchoscopy.	ARMS	*EGFR* 21 L858R	Unable to obtain pathology
7	37/F	No	Patient with acute pericardial tamponade, and intolerance to bronchoscopy and not suitable for lung puncture. Pericardial effusion cytology was negative.	NGS ARMS	*EGFR* 21 L858R (1.617%) *EGFR* 21 L858R	Adenocarcinoma NGS:*EGFR* 21L858R
8	85/F	Yes	Small intrapulmonary lesions those were not successfully amenable to transthoracic needle biopsy. Pleural fluid cytology was negative.	ARMS	*EGFR* 21 L858R	Unable to obtain pathology
9	66/F	No	Very poor performance status limited tolerance to invasive biopsy.	ARMS	*EGFR* 21 L858R	Unable to obtain pathology
10	82/M	Unknown	Very poor performance status limited tolerance to invasive biopsy.	ARMS	*EGFR* 19 del	Unable to obtain pathology
11	68/F	Unknown	ECOG = 3. Lesions not amenable to lung puncture given anatomic location, and intolerance to bronchoscopy.	ARMS	*EGFR* 19 del	Unable to obtain pathology
12	70/M	Yes	ECOG = 3. The clotting time is obviously prolonged and there is a risk of bleeding during the invasive operation.	ARMS	*EGFR* 21 L858R	Unable to obtain pathology
13	73/M	Yes	Patient has multiple brain metastases and accompanied by intracranial hypertension and intolerance to bronchoscopy. Lesions not amenable to lung puncture given anatomic location.	ARMS	*EGFR* 19del	Unable to obtain pathology
14	58/F	No	ECOG = 4, Patient has multiple brain metastases and accompanied by intracranial hypertension and intolerance to bronchoscopy. Lesions not amenable to lung puncture given anatomic location.	NGS	*EGFR* p.T790M (0.438%), *EGFR* p.G719A (9.927%), *EGFR* p.L861 (9.792%), *EGFR* 19 747_752 del (0.5%)	Poorly differentiated carcinoma Tissue specimen ARMS: *EGFR* 18 G719X, *EGFR* 21 L861Q
15	71/M	Yes	No malignant cells were found by bronchoscopy in other hospital. Pneumothorax, ECOG = 3, intolerance to bronchoscopy again. Lesions not amenable to lung puncture given anatomic location.	ARMS	*EGFR* 21 L858R	Unable to obtain pathology
16	66/F	No	Poor performance status precluded invasive biopsy.	ARMS	*EGFR* 21 L858R	Unable to obtain pathology
17	55/M	Yes	ECOG = 4. Patient has multiple brain metastases and accompanied by intracranial hypertension and symptoms of pericardial tamponade, and intolerance to bronchoscopy. Pericardial effusion cytology was negative. Lesions not amenable to lung puncture given anatomic location.	NGS	*EGFR* 19del (3.3825%) (ddPCR *EGFR* 19 746‐750 del)	Adenocarcinoma NGS:*EGFR* 19 del
18	49/M	Yes	The patient unable to tolerance bronchoscopy due to severe vertebral metastases, and lesions not amenable to lung puncture given anatomic location.	NGS	*EGFR* 19del (21.9575%) (ddPCR *EGFR* 19 746‐750 del)	Adenocarcinoma NO detection of gene due to financial condition
19	65/M	Yes	The patient intolerance to bronchoscopy and lung puncture due to severe arrhythmia and massive hemoptysis.	ARMS	*EGFR* 21 L858R	Unable to obtain pathology
20	78/M	Yes	The patient unable to tolerance bronchoscopy due to severe vertebral metastases. Lesions not amenable to lung puncture given anatomic location.	ARMS	*EGFR* 19del	Unable to obtain pathology
21	82/M	Yes	The patient intolerance to bronchoscopy due to respiratory failure and cerebral infarction. Lesions not amenable to lung puncture given anatomic location.	NGS	Wild type	
22	80/F	No	Patient has multiple brain metastases and accompanied by intracranial hypertension and intolerance to bronchoscopy. Lesions not amenable to lung puncture given anatomic location.	NGS	Wild type	
23	59/M	Yes	Patient has multiple brain metastases and accompanied by intracranial hypertension and massive hemoptysis, and intolerance to bronchoscopy. Lesions not amenable to lung puncture given anatomic location.	NGS	Wild type	
24	70/M	Yes	Paraplegia due to bone metastases. Lesion not amenable to transthoracic biopsy and lung puncture given anatomic location, patient unable to tolerate bronchoscopy.	NGS	Wild type	
25	66/M	Yes	Massive hemoptysis, not suitable for lung puncture or bronchoscopy	NGS	Wild type	
26	67/M	Yes	Paraplegia due to bone metastases. Lesion not amenable to transthoracic biopsy and lung puncture given anatomic location, patient unable to tolerate bronchoscopy.	NGS	Wild type	
27	78/M	Yes	Poor performance status precluded invasive biopsy. Lesions not amenable to lung puncture given anatomic location.	NGS	Wild type	
28	55/F	Yes	Patient has multiple brain metastases and accompanied by intracranial hypertension and intolerance to bronchoscopy. Lesions not amenable to lung puncture given anatomic location.	NGS	Wild type	
29	38/M	Yes	Paraplegia due to bone metastases. Lesion not amenable to transthoracic biopsy and lung puncture given anatomic location, patient unable to tolerate bronchoscopy.	NGS	Wild type	
30	58/M	Yes	Patient has multiple brain metastases and accompanied by intracranial hypertension and intolerance to bronchoscopy. Lesions not amenable to lung puncture given anatomic location.	NGS	Wild type	

Abbreviations: ARMS, Amplification Refractory Mutation System; ECOG, Eastern Cooperative Oncology Group; NGS, next‐generation sequencing; VAF, variant allele frequency.

a Four patients subsequently underwent gene testing on tissue specimens, and three of them were consistent with peripheral blood tests. NGS results in one case of peripheral blood: *EGFR* p.T790M (0.438%), *EGFR* p.G719A (9.927%), *EGFR* p.L861Q (9.792%), and *EGFR* 19 p747_752del (0.5%) mutation. The results of ARMS analysis of tissue samples showed G719X mutations in exon 18 and L861Q mutations in exon 21 of *EGFR*.

## 4 DISCUSSION

As EGFR‐targeted therapy has become the first‐line treatment for patients with advanced NSCLC with sensitizing *EGFR* mutations, the need for molecular profiling at the time of initial diagnosis is critically important. Unfortunately, many patients with advanced NSCLC patients are unable to tolerate tissue biopsies due to poor performance status and comorbidities precluding an invasive operation. This study showed that liquid biopsies using ctDNA can be used for initial diagnosis in those patients with suspected advanced NSCLC who are unable to get tissue biopsies.

Studies have shown that ctDNA analysis is able to detect driver *EGFR* mutations in patients' plasma with high sensitivity and specificity, correlating with clinical outcomes including ORR, PFS, and OS.^4^, ^10^, ^11^ In September 2014, the European Medicines Agency (EMA) recommended the use of plasma ctDNA for genotyping in cases where tissue sample was not available. The US Food and Drug Administration (FDA) approved the cobas^®^ *EGFR* Mutation Test v2 to detect the presence of specific NSCLC mutations (exon 19 deletion or exon 21 [L858R] substitution) in patients' blood to determine who would be candidates for treatment with erlotinib, as well as to detect patients with T790M mutations who would benefit from osimertinib. In China, an expert consensus statement recognized ctDNA as an appropriate alternative for *EGFR* mutation testing, NSCLC subtype diagnosis, and treatment monitoring when tumor tissue biopsy is infeasible.^12^ Studies have shown that in ctDNA *EGFR* mutant patients treated with EGFR TKIs, ORR, PFS, and OS are similar to that in patients with *EGFR* mutations detected in tumors. In a study of advanced NSCLC patients treated with gefitinib, Douillard et al reported an ORR of 76.9% and PFS of 10.2 months based on plasma‐detected *EGFR* mutants.^10^ The independent review committee assessed PFS to be 11 months for ctDNA *EGFR* mutant patients treated with gefitinib in the ENSURE study.^13^, ^14^ In our study, the ORR, DCR, and PFS for the treatment group were 90%, 100%, and 10 months, respectively. This suggests that patients without pathologic diagnosis had a similar clinical outcome and prognosis as patients in groups where histopathologic diagnosis was obtained. In addition to improvement in survival, all 20 patients in the treatment group showed improvement in their performance status. One patient had a complex *EGFR* mutation including T790M. Since he was previously untreated, we believe that this was a germline mutation as has been reported in other studies.^15^ Interestingly, this patient responded to first‐generation EGFR TKIs.

This study has certain limitations. First, this is not a randomized study. Future multicenter randomized clinical trials with large sample sizes are needed to validate our results. Second, the detection rate is contingent on biological factors such as tumor load and degree of vascularization of the tumor as this will impact the amount of ctDNA shed into the circulation. We used serum tumor markers as a diagnostic aid as outlined by Chinese national guidelines. We acknowledge that there are no validated serum tumor markers for lung cancer. However, we used them to supplement imaging data and clinical presentation, in order to increase the sensitivity of diagnosis. This was performed to ensure a high degree of suspicion before starting EGFR TKIs in patients who lacked histopathologic confirmation. Although germline *EGFR* T790M mutations have been reported in the literature, to our knowledge there are no reported germline sensitizing *EGFR* mutations which could have confounded our results.^15^

Despite these limitations, this is the first prospective study assessing the feasibility of utilizing ctDNA alone in detecting *EGFR* mutations in patients with suspected lung cancer that lack histologic diagnosis. Remon et al enrolled 116 pretreated advanced NSCLC patients (1 treatment‐naive), and performed NGS‐based plasma genotyping.^16^ In patients with previously unknown molecular profiles, two patients were treated with EGFR TKIs. The authors concluded that plasma genotyping was a feasible alternative to tumor genotyping in patients, negating the need for an invasive tumor tissue biopsy. However, unlike our patient population, these patients had prior histologic confirmation of their NSCLC. Guidelines exist for the management of occult primary tumors, however, we studied a group of patients with absolutely no histologic confirmation of malignancy. About 10%‐15% of advanced NSCLC patients are unable to receive treatment because tumor tissue samples are unavailable.^17^ Studies have shown a high degree of concordance of cfDNA analysis with tissue testing.^18^ In the IFUM study, the mutation status of *EGFR* in blood and matched tissues of 652 patients with NSCLC cancer was detected by ARMS. The concordance, sensitivity, and specificity of *EGFR* mutation detection in blood and tissue samples were 94.3%, 65.7%, and 99.8%, respectively. Concordance of ctDNA by NGS with matched tissues ranged from 60% to 86% in patients with advanced lung cancer. And sensitivity was 87%, 100% for 19 Del, exon 21 L858R. Since *EGFR* mutations are reasonably specific for NSCLC, with frequencies as high as 58% in Asian patients with lung adenocarcinoma,^19^ it is rational to treat patients based on the detection of *EGFR* mutations in the setting of clinically suspected advanced lung cancer without pathologic diagnosis. For these patients, plasma molecular profiling is an important alternative to establish diagnosis and guide further treatment. Incorporating this key information into other available clinical data including clinical presentation and imaging, may allow patients with advanced lung cancer to benefit from targeted therapy and gain significant improvement in survival.

## 5 CONCLUSIONS

We conducted a prospective study of 30 Chinese patients with suspected advanced lung cancer, who were unable to undergo a biopsy for initial diagnosis due to comorbidities or poor performance status. Patients with plasma *EGFR* sensitizing mutations were treated with first‐generation EGFR TKIs. This is the first prospective study showing that ctDNA genotyping provides a feasible diagnostic approach for frail lung cancer patients who are unable to undergo biopsy, which subsequently leads to EGFR‐targeted therapy resulting in improved outcomes in a subgroup of patients.

## CONFLICT OF INTERESTS

The authors declare that they have no competing interests.

## AUTHORS' CONTRIBUTIONS

Qinfang Deng, Qiyu Fang, Hui Sun, and Aditi P. Singh contributed equally to this work. Haiying Cheng and Songwen Zhou conceived, designed the study, and summarized the results. Qinfang Deng, Qiyu Fang, and Hui Sun collected the human sample and completed gene sequencing of ctDNA. Qinfang Deng, Qiyu Fang, Aditi P. Singh, Mariam Alexander, and Shenduo Li performed analysis and interpretation of all data and drafted the article. All authors read and approved the final manuscript.

## ETHICS APPROVAL AND CONSENT TO PARTICIPATE

This study was approved by the Human Research Ethics Committee of Union Affiliated to Shanghai Pulmonary Hospital, Tongji University School of Medicine.

## CONSENT FOR PUBLICATION

Not applicable.

## Data Availability

The datasets used and analyzed during the current study are available from the corresponding authors on reasonable request.
