# Supplementary material for: Detection of plasma EGFR mutations for personalized treatment of lung cancer patients without pathologic diagnosis
Source: Cancer Med. 2020 Jan 28;9(6):2085–95. doi: 10.1002/cam4.2869 (PMC7064093; doi:10.1002/cam4.2869)
Supplement: Supplementary file 1 [file CAM4-9-2085-s001.docx]

Supplementary Table 1 (Gene list)

| *ABCB1* | *ABL1* | *ACVR1B* | *AKT1* | *AKT2* | *ALK* | *APC* |
| --- | --- | --- | --- | --- | --- | --- |
| *APOB* | *AR* | *ARAF* | *ARID1A* | *ARID2* | *ATM* | *AXIN1* |
| *BAP1* | *BCL2* | *BRAF* | *BRCA1* | *BRCA2* | *CARD11* | *CBL* |
| *CCND1* | *CCND2* | *CCND3* | *CCNE1* | *CD274* | *CDA* | *CDH1* |
| *CDK4* | *CDK6* | *CDKN2A* | *CDKN2B* | *CFTR* | *CHEK2* | *CREBBP* |
| *CRLF2* | *CSF1R* | *CTNNB1* | *CYP19A1* | *CYP2C8* | *CYP2D6* | *DDR2* |
| *DNMT1* | *DNMT3A* | *DPYD* | *DYNC2H1* | *EGFR* | *EP300* | *ERBB2* |
| *ERBB3* | *ERBB4* | *ERCC1* | *ERCC2* | *ESR1* | *EZH2* | *FANCA* |
| *FANCC* | *FBXW7* | *FGFR1* | *FGFR2* | *FGFR3* | *FGFR4* | *FLT3* |
| *GATA2* | *GATA3* | *GNA11* | *GNAQ* | *GNAS* | *GSTM1* | *GSTP1* |
| *GSTT1* | *H3F3A* | *HRAS* | *IDH1* | *IDH2* | *IL2RA* | *IRF5* |
| *JAK1* | *JAK2* | *JAK3* | *KDM6A* | *KDR* | *KEAP1* | *KIT* |
| *KLF4* | *KMT2D* | *KRAS* | *LRP1B* | *MAP2K1* | *MAP2K2* | *MAP3K1* |
| *MCL1* | *MED12* | *MET* | *MLH1* | *MPL* | *MS4A1* | *MSH2* |
| *MSH6* | *MTHFR* | *MTOR* | *MYCN* | *MYD88* | *NEFH* | *NF1* |
| *NF2* | *NFE2L2* | *NOTCH1* | *NQO1* | *NRAS* | *NTRK1* | *NUDT15* |
| *PBRM1* | *PDGFB* | *PDGFRA* | *PDYN* | *PGR* | *PIK3CA* | *PIK3R1* |
| *PMS1* | *PMS2* | *PPM1E* | *PPP2R1A* | *PTCH1* | *PTEN* | *PTPN11* |
| *RB1* | *RET* | *RHOA* | *ROS1* | *RRM1* | *RUNX1* | *SF3B1* |
| *SLCO1B1* | *SMAD4* | *SMARCA4* | *SMARCB1* | *SMO* | *SPOP* | *SPTA1* |
| *STAG2* | *STK11* | *TEKT4* | *TP53* | *TPMT* | *TSC1* | *TSC2* |
| *TSHR* | *TYMS* | *U2AF1* | *UGT1A1* | *USH2A* | *VHL* | *WT1* |
| *XPC* | *XRCC1* |  |  |  |  |  |
